# Altered Prefrontal Dynamic Functional Connectivity in Vascular Dementia During Olfactory Stimulation: An fNIRS Study

**DOI:** 10.3390/bioengineering12111172

**Published:** 2025-10-28

**Authors:** Sungchul Kim, Seonghyun Kim, Seung Ha Hwang, Jaewon Kim, Ho Geol Woo, Dong Keon Yon

**Affiliations:** 1 Institute for Artificial Intelligence, N.CER, Gwangju 61005, Republic of Korea; 2 Department of Biomedical Science and Engineering, Gwangju Institute of Science and Technology (GIST), Gwangju 61005, Republic of Korea; 3 Center for Digital Health, Medical Science Research Institute, Kyung Hee University Medical Center, Kyung Hee University College of Medicine, Seoul 02447, Republic of Korea; 4 Department of Neurology, Kyung Hee University Medical Center, Kyung Hee University College of Medicine, Seoul 02447, Republic of Korea

**Keywords:** functional near-infrared spectroscopy, vascular dementia, olfactory stimulation, dynamic functional connectivity

## Abstract

In this study, we employed functional near-infrared spectroscopy (fNIRS) to explore dynamic functional connectivity (dFC) responses to olfactory stimulation in thirteen healthy control participants and seven patients with vascular dementia (VD). Participants underwent five rest and odor exposure cycles, and dFC was estimated using a sliding window correlation approach. The healthy control group exhibited limited changes, while the VD group exhibited more extensive fluctuations in both oxy- and deoxyhemoglobin dFC across multiple regions during several stimulation periods. Between-group analyses revealed differences, particularly during olfactory stimulation, with moderate to large effect sizes. These preliminary findings suggest that olfactory-evoked dFC may reflect altered brain network dynamics in VD and could potentially serve as a non-invasive, accessible tool to help understand vascular dementia.

## 1. Introduction

Dementia, which is a neurodegenerative condition characterized by progressive cognitive deterioration, represents a significant public health challenge for aging populations globally. The prevalence of dementia is gradually rising, along with the socioeconomic burden it entails. Vascular dementia (VD), which is a primary contributor to dementia alongside Alzheimer’s disease, arises from brain tissue damage resulting from cerebrovascular illness and accounts for a large proportion of all dementia cases [1,2]. The pathogenic mechanisms and clinical symptoms of VD often overlap with those of other neurodegenerative disorders, including Alzheimer’s disease and Parkinsonian syndromes, making precise differential diagnosis, particularly in the early stages, a crucial challenge. Therefore, there is an immediate necessity to establish novel, objective, and non-invasive biomarkers for the early detection and diagnosis of VD [3].

The importance of olfactory function as an early indicator of neurodegenerative disorders has recently been highlighted. In particular, within the field of Alzheimer’s disease, the relationship between olfactory dysfunction and disease progression has been investigated extensively [4,5]. Furthermore, by employing neuroimaging and neurophysiological techniques such as functional magnetic resonance imaging (fMRI), electroencephalography (EEG), and functional near-infrared spectroscopy (fNIRS), numerous studies have sought to measure brain responses to olfactory stimulation [6,7,8,9,10,11]. These approaches have enabled researchers to identify the characteristic patterns of brain activation in patients with Alzheimer’s disease and mild cognitive impairment, thereby establishing objective biosignals that may serve as potential biomarkers. Beyond Alzheimer’s disease, olfactory impairment is widely recognized as one of the earliest and most prevalent non-motor features of Parkinson’s disease, often preceding the onset of motor symptoms by several years [12,13,14]. Recent studies using neuroimaging modalities such as MRI and EEG have investigated the neural correlates of olfactory dysfunction in Parkinson’s disease, demonstrating both structural and functional alterations within olfactory-related networks [15,16]. These findings support that olfactory dysfunction has been extensively and objectively investigated in neurodegenerative diseases, particularly Parkinson’s and Alzheimer’s diseases.

In contrast, although clinical reports have consistently indicated that patients with VD exhibit impaired olfactory function, physiological studies aimed at validating this phenomenon objectively through brain signal measurements remain scarce [17,18,19]. Unlike in Alzheimer’s disease research, there is a lack of objective data establishing a direct link between olfactory dysfunction and alterations in brain function in VD. To address this gap, this study employed fNIRS to investigate differences in brain activation between patients with VD and a healthy control (HC) group during olfactory stimulation.

More recently, neuroimaging research has shifted focus from static to dynamic interactions, with growing interest in dynamic functional connectivity (dFC). Unlike conventional static analysis, dFC captures transient changes in connectivity pattern among brain regions, offering a refined tool to understand the temporal instability and reconfigurations of brain networks associated with neurodegenerative disorders. Studies utilizing dFC have revealed that neurodegenerative disorders, including Alzheimers’s disease and schizophrenia, are characterized by temporal instability in brain network connectivity and the presence of transient states of dysconnectivity, which correlate with cognitive and clinical symptoms [20,21].

This study aimed to compare dFC in response to specific olfactory stimulation between patients with VD and HC participants by quantitatively using fNIRS in a small study population. Conducted as a pilot study, it seeks to provide preliminary and quantitative data on differences in prefrontal activation patterns. The findings are expected to deepen the understanding of VD-related cortical dynamics and provide a basis for further investigations using more extensive and definitive study designs.

## 2. Methods

### 2.1. Participants

To investigate differences in olfactory responsiveness between healthy individuals and patients with VD, we recruited a group of clinical study participants. Cognitive function was assessed in all participants using the Mini-Mental State Examination (MMSE). The healthy group comprised 13 cognitively normal volunteers (8 females, 5 males; median age = 74 years, interquartile range [IQR]: 71–76 years; median MMSE score = 27, IQR: 25–29) who underwent voluntary health check-ups at the Gwangju Dementia Cohort Center and were enrolled with approval from the Institutional Review Board of the Gwangju Institute of Science and Technology (20210115-HR-58-01-02). The VD group included seven patients (three females, four males; median age = 77 years, IQR: 67–84 years; median MMSE score = 18, IQR: 5–18) diagnosed with VD at the Department of Neurology, Kyung Hee University Medical Center, with ethical oversight for this observational study being provided by the Institutional Review Board of Kyung Hee University (KHSIRB-24-427). Detailed clinical and demographic information for the patients with VD is presented in Table 1, and individual medication details and dosage schedules are provided in Supplementary Materials Table S1.

### 2.2. Data Acquisition

**Figure 1:**
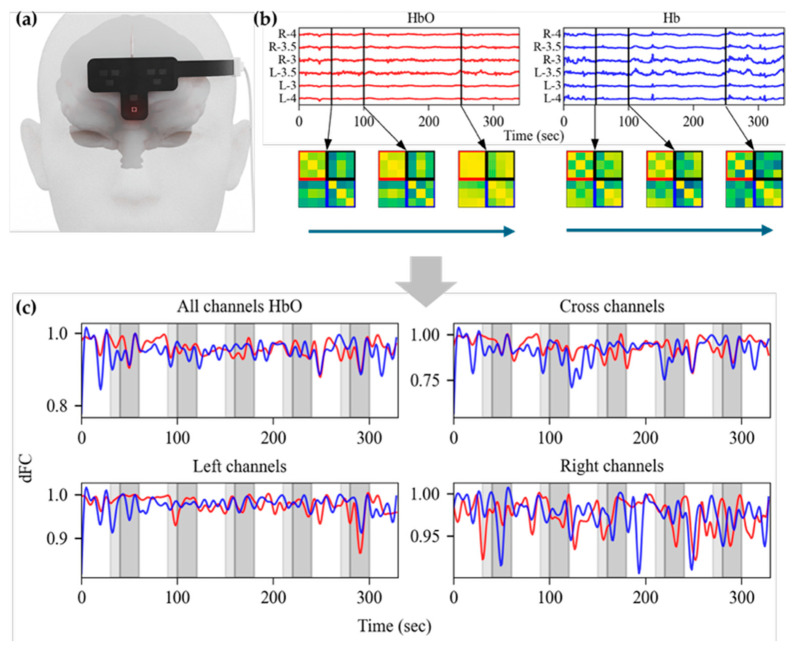
dFC analysis pipeline. (**a**) fNIRS probe placement for dFC measurement. (**b**) Functional connectivity calculation. Following short-channel regression, FC was calculated from six channels, yielding 330 smoothed FC maps. In the FC maps, the red (second quadrant), black (first quadrant), and blue (fourth quadrant) boxes indicate connectivity within the right channels, cross channels, and left channels, respectively. (**c**) Calculated dFC. Vectorized FC values for each ROI were converted into dFC values by computing the correlation coefficients between adjacent FC maps. In the bottom graph, the red and blue lines represent HbO and Hb, respectively. Light-gray shaded regions indicate the periods during which the sliding window included olfactory stimulation, whereas dark-gray areas represent the actual olfactory stimulation intervals.

During olfactory stimulation, fNIRS signals were recorded using a seven-channel NIRS system (N1, N.CER Co., Gwangju, Republic of Korea) at a sampling rate of 10 Hz. Light sources were positioned above the eyebrows, and detectors were placed at 3, 3.5, and 5 cm from the central source. In this study, our probe set was positioned approximately at FP1 and FP2 according to the international 10–20 system, covering the prefrontal cortex including the superior part of the orbitofrontal cortex (Figure 1a). A short-separation channel was located 1 cm from the central source. Optical density (OD) was calculated from the raw signals at each wavelength according to the modified Beer–Lambert law. The OD signals were preprocessed using a bandpass filter (0.001–0.5 Hz), and changes in oxyhemoglobin (HbO) and deoxyhemoglobin (Hb) concentrations were subsequently estimated. To minimize superficial physiological noise, the short-separation channel was applied to all other channels for regression analysis.

Olfactory stimulation consisted of five consecutive cycles of 40 s rest periods and 20 s odor exposure periods, followed by an additional 40 s rest period after the final stimulus. During the stimulation phase, participants were presented with odorants via sniffer sticks in the following sequence: odorless, orange, peppermint, leather, and odorless again.

### 2.3. Data Processing

In this study, we analyzed dFC during the olfactory stimulation protocol. Following the approach proposed by Zhen Li [21], dFC was estimated using the sliding window correlation method. A window length of 10 s with a step size of 0.1 s was applied, resulting in 3300 FC maps per subject. Every 10 consecutive FC maps were averaged to reduce short-term fluctuations, resulting in 330 smoothed FC maps. The FC maps were then divided into four regions of interest (ROIs): all channels, left channels, right channels, and cross channels. For each ROI, the smoothed FC maps were vectorized, and correlation coefficients were computed, yielding a time series of 330 values that quantified the temporal variability of FC.

### 2.4. Statistical Analysis

For each time point in the ROI-specific dFC time series, group differences were assessed using the Mann–Whitney exact test. Additionally, Cliff’s δ, which is a nonparametric effect size measure, was calculated to quantify the magnitude of group differences. Statistical significance was defined as *p* < 0.05, and a large effect size was defined as |δ| > 0.474. Furthermore, bootstrap resampling with 500 iterations was applied to estimate the 95% confidence intervals, and effects were considered meaningful if the lower bound of the CI exceeded |δ| > 0.33.

Within-group analyses were conducted to examine whether dFC changed before and after olfactory stimulation. For each group, the mean dFC values were calculated by averaging the 30 s period preceding stimulus onset and the 30 s period following stimulus onset as well as the 30 s periods before and after stimulus offset. The statistical significance of these within-group differences was evaluated using the Wilcoxon signed-rank test. Effect sizes were quantified using rank-biserial correlation, and bootstrap resampling with 500 iterations was applied to estimate the 95% confidence intervals. Effects were considered robust when the lower bound of the confidence interval exceeded 0.3. A visualization of the procedure used for dFC analysis is presented in Figure 1.

## 3. Results

**Figure 2:**
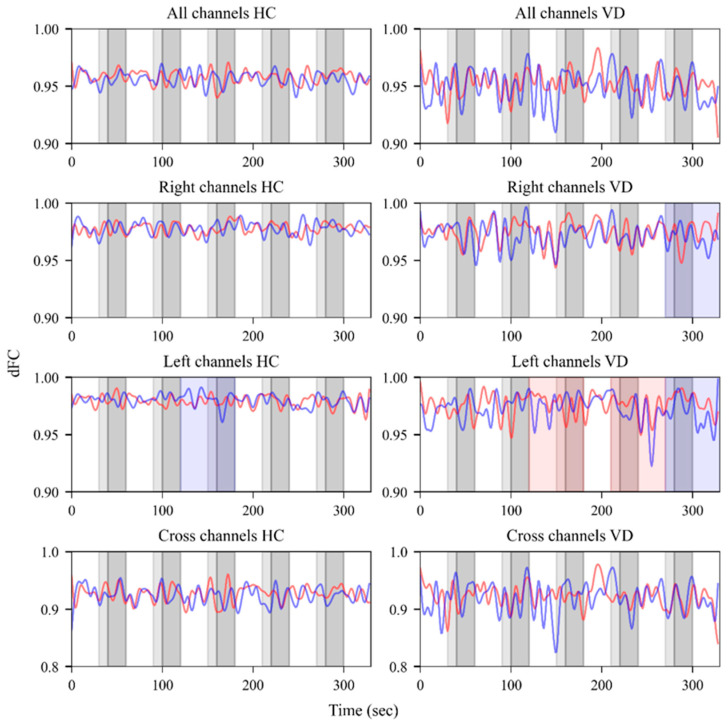
dFC for all channels, right channels, left channels, and cross channels (top to bottom). The left panels represent the HC group and the right panels represent the VD group. Red lines indicate the mean of HbO dFC and blue lines indicate the mean of Hb dFC. In each graph, light-gray shaded areas represent periods during which the sliding window overlapped with olfactory stimulation onset and dark-gray shaded areas indicate the actual olfactory stimulus periods. Red-shaded regions indicate HbO dFC showed statistically significant differences, and blue-shaded regions indicate significant differences in Hb dFC (*p* < 0.05), where the lower bound of the 95% confidence interval for the rank-biserial correlation exceeded 0.3.

Within-group analyses were conducted to evaluate changes in dFC before and after olfactory stimulation, as shown in Figure 2. In the HC group, significant changes were observed in Hb dFC within the left-channel ROI during the third olfactory stimulus, whereas no notable changes were detected in dFC for all channels, cross channels, or right-channel ROIs.

In the VD group, no significant changes were observed in dFC for all channels or cross channels. However, significant changes in Hb dFC were observed in the right-channel ROI during the last olfactory stimulus and immediately after stimulus offset. Within the left channels, HbO dFC exhibited significant changes during the third stimulus (pre- and during stimulation) and fourth stimulus (during and post-stimulation), whereas Hb dFC exhibited significant changes during and after the last stimulus.

All observed significant changes exhibited a pattern in which the dFC values were maintained before decreasing, indicating substantial fluctuations in functional connectivity among the channels within each ROI. These findings suggest that olfactory stimulation induced pronounced alterations in functional connectivity in the VD group.

**Figure 3:**
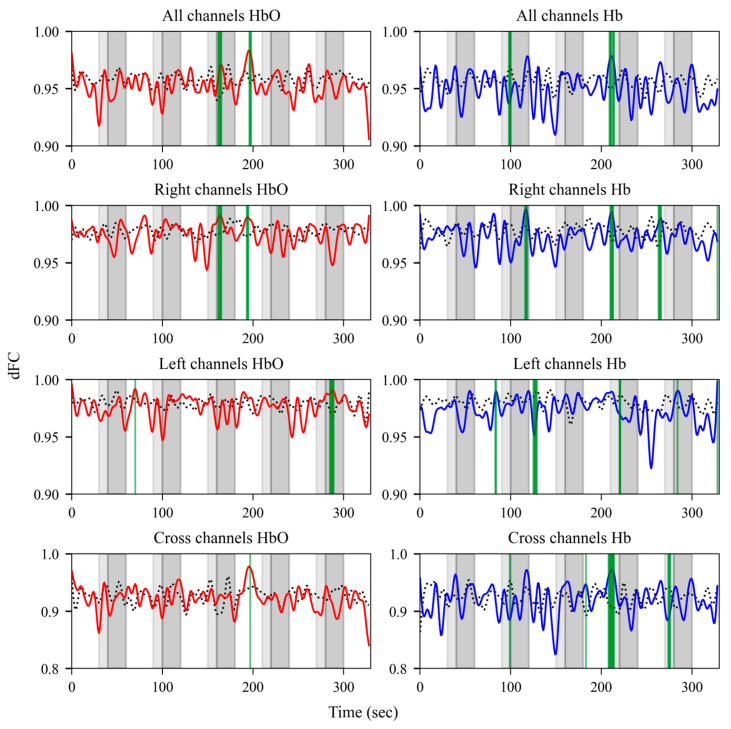
Group differences in dFC across multiple ROIs. The panels present dFC for all right, left, and cross channels (top to bottom). The left panels represent HbO dFC and right panels represent Hb dFC. Dashed black lines correspond to the mean of the HC group, while solid red and blue lines correspond to the mean of the VD group. Light-gray shaded areas indicate periods when the sliding window overlapped with the onset of olfactory stimulation and dark-gray shaded areas indicate the actual stimulus period. Green-shaded vertical regions indicate dFC showing statistically significant group differences (*p* < 0.05), where the lower bound of Cliff’s δ 95% confidence interval exceeded 0.33.

Figure 3 presents the group differences in dFC across multiple ROIs. For HbO, significant differences were detected in all channels and right channels during the third olfactory stimulus, while the left channels exhibited notable differences during the last stimulus. In contrast, Hb dFC exhibited significant group differences around the second olfactory stimulus across most channels. Specifically, significant differences were observed in all channels during the fourth stimulus, in the right channels during the fourth and fifth stimuli, in the left channels during the fifth stimulus, and in the cross channels during the fourth and fifth stimuli.

## 4. Discussion

This study aimed to investigate differences in prefrontal dFC changes between patients with VD and an HC group in response to olfactory stimulation using fNIRS. dFC provides insights into the temporal variability of brain networks that cannot be captured using static functional connectivity measures. This dynamic perspective enables the detection of olfactory-evoked signal changes, which may help differentiate patients with VD who have impaired olfactory function from healthy individuals. The results of our analysis support this expectation. Our findings demonstrate that olfactory stimulation induced both within-group changes and between-group differences in dFC. Within the HC group, only limited changes were observed, primarily in Hb within the left-channel ROI during the third stimulus, suggesting relatively stable functional connectivity in response to olfactory stimulation. In contrast, the VD group exhibited more widespread and pronounced alterations, with significant fluctuations in both HbO and Hb across the left and right channels during multiple stimuli, indicating the greater sensitivity of functional connectivity to olfactory stimulation in these patients (Figure 2). Between-group comparisons indicated that participants with VD exhibited larger changes in dFC compared with the HC group (Figure 3). Notably, significant group differences in dFC were observed during the olfactory stimulation periods. These findings suggest that olfactory-evoked dFC may serve as an indicator of functional connectivity changes in VD and could potentially function as an auxiliary tool to assist in its clinical diagnosis.

In this study, our fNIRS probe set was positioned over the prefrontal cortex, covering the upper part of the orbitofrontal cortex, which together play key roles in higher-order olfactory processing. The neural processing of olfactory information differs from other sensory modalities, involving direct projections from the olfactory bulb to the primary olfactory cortex including the piriform cortex. From the olfactory cortex, olfactory information rapidly disseminates to the secondary olfactory network, which includes important regions such as the orbitofrontal cortex, prefrontal cortex, insula, amygdala, and hippocampus [22]. The orbitofrontal and prefrontal cortex are critical for higher-order olfactory functions such as odor identification, discrimination, decision-making, and hedonic evaluation. Together, these regions form a distributed network responsible for the complex interpretation and emotional valence of odors, and the integrity of this network is often compromised early in neurodegenerative diseases.

These findings highlight the potential of olfactory-evoked dFC as an indicator of functional network alterations in VD. However, the mechanisms underlying the greater changes in functional connectivity observed in dementia remain unclear. Considering that vascular dementia frequently coexists with other neurodegenerative pathologies, including tauopathies, comorbidity may contribute to the observed variability in functional connectivity. In our study population, some patients with vascular dementia presented with comorbid conditions such as hypertension, hyperlipidemia, and diabetes, which are also commonly observed in vascular forms of corticobasal syndrome. This overlap suggests that vascular pathogenesis may influence tau-related neurodegenerative processes [23]. Further research is warranted to elucidate these potential mechanisms more precisely.

For Alzheimer’s disease, numerous studies have actively employed modalities such as EEG, fMRI, and fNIRS. Since olfactory stimulation was reported to be associated with cognitive decline, olfactory-based analyses using these techniques have been shown to be useful for distinguishing mild cognitive impairment from Alzheimer’s disease [7,8,9,10,11]. Similarly, for Parkinson’s disease, neuroimaging and neurophysiological approaches, including MRI and EEG, have been used to investigate olfactory dysfunction, demonstrating both structural and functional alterations within olfactory-related networks [15,16]. In contrast, for VD, some studies have reported EEG-based analyses, and positron emission tomography-computed tomography or fMRI has been used to compare brain function between patients with VD and HC individuals [24,25]. However, research on olfactory stimulation in VD has largely remained limited to diagnostic questionnaires [18,19]. To the best of our knowledge, this study represents an initial attempt to apply a biosignal-based analytical approach to characterize olfactory-related brain responses in patients with VD quantitatively.

Despite the relatively small sample size of this study, statistically significant differences between groups were observed, which were evaluated using the Mann–Whitney exact test (*p* < 0.05), a nonparametric method chosen to account for the small sample size and non-normal data. Effect sizes were calculated using Cliff’s δ and supplemented with bootstrap resampling to estimate the 95% confidence intervals; effects were considered meaningful when the lower bound of the CI exceeded 0.33, indicating at least a medium effect. Nevertheless, the limited number of participants necessitates cautious interpretation.

Because of the small sample size in this study, participants were not stratified by medication type, nor was medication status included as a covariate. However, future studies with larger cohorts should consider the potential influence of pharmacological treatments—such as serotonergic agents or cholinesterase inhibitors—on prefrontal cortical activation, as these medications may modulate fNIRS responses. Given that dementia encompasses multiple subtypes, including Alzheimer’s disease, VD, and dementia with Lewy bodies, further research should also examine subtype-specific olfactory hemodynamic responses to clarify potential diagnostic signatures.

Notably, olfactory stimulation combined with biosignal analysis as a screening tool for dementia has inherent limitations. For example, accuracy may be affected by transient conditions such as rhinitis or the common cold, and this method cannot precisely localize the cerebrovascular lesions underlying VD [26]. Nevertheless, our findings are clinically meaningful, as they highlight the potential of a rapid, objective, and cost-effective tool that could serve as an additional test to assist in understanding the diagnosis of VD. Future work should adopt longitudinal designs to test whether the identified hemodynamic patterns can predict subsequent cognitive decline, and comparative studies across different dementia subtypes are necessary to establish discriminative validity.

## 5. Conclusions

This study demonstrated that olfactory stimulation induced limited dFC changes in HC subjects but more widespread and pronounced alterations in patients with VD, as observed in both HbO and Hb across multiple regions. Significant between-group differences were consistently observed during the stimulation periods, supporting the sensitivity of olfactory-evoked dFC to VD-related changes. These findings suggest that fNIRS-based dFC analysis may provide a rapid, objective, and cost-effective tool to assist in differentiating VD from healthy aging. Despite the small sample size of this study, the clinical relevance of our approach is evident, and larger, longitudinal studies are warranted to validate its robustness and explore its applicability across dementia subtypes.

## Figures and Tables

**Table 1:** Demographics, comorbidities, and status of targeted medication use in patients with VD.

Patient ID	Age	Sex	Hypertension	Diabetes Mellitus	Hyperlipidemia	Serotonergic Drugs	Cholinesterase Inhibitors
1	60	M	Yes	No	No	None	None
2	60	M	Yes	No	No	Motilitone	None
3	77	M	Yes	No	Yes	None	None
4	83	F	Yes	No	Yes	None	None
5	85	M	Yes	No	Yes	None	None
6	86	F	Yes	Yes	Yes	None	None
7	75	F	Yes	Yes	Yes	None	None

## Data Availability

The data cannot be made publicly available upon publication due to legal restrictions preventing unrestricted public distribution. The data that support the findings of this study are available upon reasonable request from the authors.

## Supplementary Materials

The following supporting information can be downloaded at: https://www.mdpi.com/article/10.3390/bioengineering12111172/s1, Table S1: Demographics, comorbidities, and status of targeted medication use including drug dosage and frequency in patients with VD.

## Abbreviations

The following abbreviations are used in this manuscript:

dFC	Dynamic functional connectivity
EEG	Electroencephalography
fMRI	Functional magnetic resonance imaging
fNIRS	Functional near-infrared spectroscopy
Hb	Deoxyhemoglobin
HbO	Oxyhemoglobin
HC	Healthy control
MMSE	Mini-Mental State Examination
OD	Optical density
ROI	Region of interest
VD	Vascular dementia
