# Supplementary material for: Altered Prefrontal Dynamic Functional Connectivity in Vascular Dementia During Olfactory Stimulation: An fNIRS Study
Source: Bioengineering (Basel). 2025 Oct 28;12(11):1172. doi: 10.3390/bioengineering12111172 (PMC12649715; doi:10.3390/bioengineering12111172)
Supplement: Supplementary file 1 [file bioengineering-12-01172-s001.zip › bioengineering-3934879-supplementary.pdf]

Supplementary Table S1. Demographics, Comorbidities, and Status of Targeted Medication Use, including drug dosage and frequency in patients with VD.

Patient ID	Age	Sex	Hypertension	Diabetes Mellitus	Hyperlipidemia	Medications (Dosage/Freq)	
1	60	M	Yes	No	No	qd	Aspirin 100, atozet 10/40, dapagliflozin 10, lanston 30
						bid	ticlopidine, sevikar 5/20, concor 2.5, metformin 500, medirac
						tid	Mucosta 100, mitilitone, sermion
2	60	M	Yes	No	No	qd	Warfarin, digoxin 0.25, atozet 10/40, concor 2.5, lanston 30, harnal-d
						bid	Metformin 500
						tid	Mucosta 100
3	77	M	Yes	No	Yes	qd	aspirin 100, Plavix, atozet 10/40, gemigliptin, metformin 500
						bid	Sevikar 5/20, concor 2.5, sermion
						tid	Mucosta 100, mukaran, baclofen 0.5t
4	83	F	Yes	No	Yes	qd	atozet 10/40, esomeprazole 40
						bid	Apixaban 2.5, sevikar 5/20
						tid	Mucosta 100, mukaran
5	85	M	Yes	No	Yes	qd	Atozet 10/40, colchine 0.6, feburic 40 0.5t
						bid	Apixaban 5, concor 5, Zyloric 100, azeptin, vimovo 500/20
						tid	Diltiazem 30, bioflor, mukaran
6	86	F	Yes	Yes	Yes	qd	Aspirin 100, Plavix, atozet 10/40, esomeprazole 40
						bid	Apixaban 2.5, carvedilol 6.25
						tid	Mucosta 100, legalon, godex
7	75	F	Yes	Yes	Yes	qd	Warfarin 5, atozet 10/40, esomeprazole 40
						bid	None
						tid	Mucosta 100, legalon, godex, acetaminophen

qd: once daily (quaque die); bid: twice daily (bis in die); tid: three times daily (ter in die).
